# Rates of Opioid Overdose Among Racial and Ethnic Minority Individuals Released From Prison

**DOI:** 10.1001/jamahealthforum.2023.4455

**Published:** 2023-12-21

**Authors:** Benjamin A. Barsky, Devon Dunn, Elizabeth A. Erdman, James R. Jolin, Meredith B. Rosenthal

**Affiliations:** 1 Interfaculty Initiative in Health Policy, Harvard University, Cambridge, Massachusetts; 2 Edmond & Lily Safra Center for Ethics, Cambridge, Massachusetts; 3 Massachusetts Department of Public Health, Boston; 4 Government and Global Health and Health Policy, Harvard College, Cambridge, Massachusetts; 5 Harvard T.H. Chan School of Public Health, Boston, Massachusetts

## Abstract

This cross-sectional study examines opioid overdose patterns by race and ethnicity among individuals released from prison in Massachusetts.

## Introduction

Increases in opioid overdoses among racial and ethnic minority individuals have outpaced those among White people in the US.^1^ Whether this trend holds for recently incarcerated people—a population disproportionately at risk of opioid overdose—is unclear.^2^ Factors like inequitable treatment access and differential exposure to fentanyl may drive disparities within this population.^3,4^ We assessed opioid overdose patterns among people released from prison in Massachusetts.

## Methods

Using data from the Massachusetts Public Health Data Warehouse, this cross-sectional study identified nonfatal opioid overdoses (NFOs) and fatal opioid overdoses (FOs) among adults released from prison between January 2014 and December 2019 (eAppendix in Supplement 1). We focused on events within 12 months of release because of elevated opioid overdose risk during that period. The Harvard School of Public Health’s institutional review board exempted the study and waived informed consent because this was not human participants research. We followed the STROBE reporting guideline.

Using logistic regression, we identified demographic (race and ethnicity [Hispanic; non-Hispanic American Indian, Asian/Pacific Islander, or other; non-Hispanic Black; or non-Hispanic White], sex, and age) and offense characteristics associated with experiencing NFOs and FOs. We analyzed data from October 2022 through October 2023 in SAS Studio, version 3.81.

## Results

Among 10 920 individuals released from prison, 6.8% and 1.3% experienced NFOs and FOs, respectively, within 12 months. The NFO rate per 10 000 releases in 2014 was 65.79, 245.7, and 892.67 for Black, Hispanic, and White people, respectively, and was 289.86 for Black (340.6% increase; annual mean [SD] increase, 87.7% [180.2%]), 474.04 for Hispanic (92.9% increase; annual mean [SD] increase, 15.6% [22.2%]), and 1123.47 for White (25.9% increase; annual mean [SD] increase, 6.1% [19.0%]) people in 2019. Fewer than 11 NFOs occurred among people of American Indian, Asian/Pacific Islander, or other race and ethnicity. Individuals who were Black (adjusted odds ratio [AOR], 0.25; 95% CI, 0.19-0.32), Hispanic (AOR, 0.41; 95% CI, 0.32-0.52), male (AOR, 0.72; 95% CI, 0.58-0.88), 55 years or older (AOR, 0.62; 95% CI, 0.44-0.88), sentenced for not clearly categorized offenses (AOR, 0.61; 95% CI, 0.47-0.79), or incarcerated for 4 or more years (AOR, 0.38; 95% CI, 0.29-0.51) were less likely to experience an NFO vs individuals who were White, female, aged 18 to 34 years, sentenced for offenses against persons, or incarcerated for less than 1 year, respectively (Table 1).

**Table 1.** Association Between NFOs Within 12 Months of Release From Prison and Demographic and Offense Characteristics, January 2014 to December 2019

Characteristic	Prison releases, No./total No. (%) (N = 10 920)		Adjusted OR (95% CI)^a^
	NFOs within 12 mo	No NFOs within 12 mo	
Total	747/10 920 (6.8)	10 173/10 920 (93.2)	NA
Year of release			
2014	98/1862 (5.3)	1764/1862 (94.7)	NA
2015	125/1899 (6.6)	1774/1899 (93.4)	NA
2016	113/1818 (6.2)	1705/1818 (93.8)	NA
2017	124/1772 (7.0)	1648/1772 (93.0)	NA
2018	153/1778 (8.6)	1625/1778 (91.4)	NA
2019	134/1791 (7.5)	1657/1791 (92.5)	NA
Race and ethnicity^b^			
Hispanic	93/2556 (3.6)	2463/2556 (96.4)	0.41 (0.32-0.52)
Non-Hispanic American Indian, Asian/Pacific Islander, or other^c^	NR^d^	98	0.52 (0.19-1.43)
Non-Hispanic Black	NR^e^	2610	0.25 (0.19-0.33)
Non-Hispanic White	575/5412 (10.6)	4837/5412 (89.4)	1 [Reference]
Documented sex			
Female	330/2370 (13.9)	2040/2370 (86.1)	1 [Reference]
Male	417/8550 (4.9)	8133/8550 (95.1)	0.72 (0.58-0.88)
Age group, y			
18-34	374/2370 (7.6)	4556/2370 (92.4)	1 [Reference]
35-44	222/3046 (7.3)	2824/3046 (92.7)	1.03 (0.86-1.23)
45-54	111/1964 (5.7)	1853/1964 (94.3)	0.79 (0.63-0.99)
≥55	40/980 (4.1)	940/980 (95.9)	0.62 (0.44-0.88)
Governing offense			
Drug	144/2440 (5.9)	2296/2440 (94.1)	0.90 (0.72-1.12)
Person	285/4332 (6.6)	4047/4332 (93.4)	1 [Reference]
Property	198/1722 (11.5)	1524/1722 (88.5)	1.14 (0.92-1.40)
Sex	26/733 (3.5)	707/733 (96.5)	0.70 (0.46-1.06)
Not clearly categorized^f^	94/1693 (5.6)	1599/1693 (94.4)	0.61 (0.47-0.79)
Length of incarceration, y			
<1	431/3446 (12.5)	3015/3446 (87.5)	1 [Reference]
1-2	103/1986 (5.2)	1883/1986 (94.8)	0.55 (0.43-0.70)
2-3	82/1794 (4.6)	1712/1794 (95.4)	0.51 (0.39-0.68)
3-4	50/1228 (4.1)	1178/1228 (95.9)	0.45 (0.32-0.62)
≥4	81/2466 (3.3)	2385/2466 (96.7)	0.38 (0.29-0.51)

Abbreviations: NA, not applicable; NFO, nonfatal opioid overdose; NR, not reported; OR, odds ratio.

^a^ Adjusted for age, governing offense, length of incarceration, race and ethnicity (Hispanic; non-Hispanic American Indian, Asian/Pacific Islander, or other; non-Hispanic Black; or non-Hispanic White), and sex.

^b^ Race and ethnicity were ascertained by self-report in the Department of Corrections data. This information was included in the analysis to identify disparities in rates of opioid overdose among racial and ethnic minority individuals released from prison in Massachusetts. Race and ethnicity information for 14 people who experienced a nonfatal overdose and 165 who did not were unknown.

^c^ The other category was not broken down further in the Department of Corrections data.

^d^ Cell counts between 1 and 10 were suppressed for data privacy.

^e^ Complementary suppression was applied for data privacy.

^f^ Includes obstruction of justice, habitual offender, prostitution, and some weapon possession crimes.

The FO rate per 10 000 releases in 2014 was 21.93, 0, and 127.52 for Black, Hispanic, and White people, respectively. In 2019, the rate was 96.62 for Black (340.6% increase; annual mean [SD] increase, 85.7% [136.5%]), 474.04 for Hispanic, and 222.47 for White (74.5% increase; annual mean [SD] increase, 29.7% [85.7%]) people. No FOs occurred among people of American Indian, Asian/Pacific Islander, or other race and ethnicity. Individuals who were Black (AOR, 0.38; 95% CI, 0.22-0.66), Hispanic (AOR, 0.47; 95% CI, 0.27-0.80), aged 18 to 34 years (AOR, 0.60; 95% CI, 0.38-0.95), or incarcerated for 4 or more years (AOR, 0.39; 95% CI, 0.21-0.74) were less likely to experience an FO vs individuals who were White, aged 45 to 54 years, or incarcerated for less than 1 year, respectively (Table 2).

**Table 2.** Association Between Fatal Opioid Overdoses Within 12 Months of Release From Prison and Demographic and Offense Characteristics, January 2014 to December 2019

Characteristic	Prison releases, No./total No. (%) (N = 10 920)		Adjusted OR (95% CI)^a^
	FOs within 12 mo	No FOs within 12 mo	
Total	140/10 920 (1.3)	10 780/10 920 (98.7)	NA
Year of release			
2014	13/1862 (0.7)	1849/1862 (99.3)	NA
2015	17/1899 (0.9)	1882/1899 (99.1)	NA
2016	37/1818 (2.0)	1781/1818 (98.0)	NA
2017	24/1772 (1.4)	1748/1772 (98.6)	NA
2018	20/1778 (1.1)	1758/1778 (98.9)	NA
2019	29/1791 (1.6)	1762/1791 (98.4)	NA
Race and ethnicity^b^			
Hispanic	18/2556 (0.7)	2538/2556 (99.3)	0.47 (0.27 to 0.80)
Non-Hispanic American Indian, Asian/Pacific Islander, or other^c^	0	102/102 (100)	<0.01 (<0.01 to >999.99)
Non-Hispanic Black	16/2671 (0.6)	2655/2671 (99.4)	0.38 (0.22 to 0.66)
Non-Hispanic White	102/5412 (1.9)	5310/5412 (98.1)	1 [Reference]
Documented sex			
Female	59/2370 (2.5)	2311/2370 (97.5)	1.32 (0.84 to 2.09)
Male	81/8550 (0.9)	8469/8550 (99.1)	1 [Reference]
Age group, y			
18-34	56/4930 (1.1)	4874/4930 (98.9)	0.50 (0.32 to 0.77)
35-44	38/3046 (1.2)	3008/3046 (98.8)	0.60 (0.38 to 0.95)
45-54	NR^d^	1926	1 [Reference]
≥55	NR^e^	972	0.46 (0.21 to 1.00)
Governing offense^f^			
Drug	30/2440 (1.2)	2410/2440 (98.8)	1.00 (0.63 to 1.60)
Person	53/4332 (1.2)	4279/4332 (98.8)	1 [Reference]
Property	31/1722 (1.8)	1691/1722 (98.2)	0.87 (0.54 to 1.39)
Sex	NR^e^	729	0.54 (0.19 to 1.51)
Not clearly categorized	NR^d^	1671	0.77 (0.45 to 1.32)
Length of incarceration, y			
<1	77/3446 (2.2)	3369/3446 (97.8)	1 [Reference]
1-2	27/1986 (1.4)	1959/1986 (98.6)	0.82 (0.50-1.35)
2-3	NR^d^	1781	0.46 (0.24-0.87)
3-4	NR^e^	1221	0.35 (0.15-0.79)
≥4	16/2466 (0.6)	2450/2466 (99.4)	0.39 (0.21-0.74)

Abbreviations: FO, fatal opioid overdose; NA, not applicable; NR, not reported; OR, odds ratio.

^a^ Adjusted for age, governing offense, length of incarceration, race and ethnicity (Hispanic; non-Hispanic American Indian, Asian/Pacific Islander, or other; non-Hispanic Black; or non-Hispanic White), and sex.

^b^ Race and ethnicity were ascertained by self-report in the Department of Corrections data. This information was included in the analysis to identify disparities in rates of opioid overdose among racial and ethnic minority individuals released from prison in Massachusetts. Race and ethnicity information for 4 people who experienced a fatal overdose and 175 who did not were unknown.

^c^ The other category was not broken down further in the Department of Corrections data.

^d^ Complementary suppression was applied for data privacy.

^e^ Cell counts between 1 and 10 were suppressed for data privacy.

^f^ Includes obstruction of justice, habitual offender, prostitution, and some weapon possession crimes.

## Discussion

People released from prison experienced increasing NFO and FO rates, which were highest among White people. However, the mean annual rate of increase for NFOs and FOs was highest among Black people followed by Hispanic people and White people.

Our findings contrast with data on FO rates in Massachusetts and the US showing rates among racial and ethnic minority individuals surpassing those among White people^1,5^ but align with data showing racial and ethnic minority individuals experienced the highest rates of increase in NFOs and FOs.^1,3,6^ Limitations were potential lack of generalizability to other formerly incarcerated groups (eg, jail) and possible misclassified causes of death.

It is urgent to provide tailored, racially equitable health care and harm-reduction services after prison release. Research is needed to determine whether factors driving population-level disparities in opioid overdose also affect formerly imprisoned people.^3^

## Notes

Supplement 1. **eAppendix.** Identifying Opioid-Related Overdose Events

Supplement 2.
Data Sharing Statement
