# Supplementary material for: Rates of Opioid Overdose Among Racial and Ethnic Minority Individuals Released From Prison
Source: JAMA Health Forum. 2023 Dec 21;4(12):e234455. doi: 10.1001/jamahealthforum.2023.4455 (PMC10739083; doi:10.1001/jamahealthforum.2023.4455)
Supplement: Supplement 1. — eAppendix. Identifying Opioid-Related Overdose Events [file jamahealthforum-e234455-s001.pdf]

Supplementary Online Content

Barsky BA, Dunn D, Erdman EA, Jolin JR, Rosenthal MB. Rates of opioid overdose among racial and ethnic minority individuals released from prison. *JAMA Health Forum*. 2023;4(12):e234455. doi:10.1001/jamahealthforum.2023.4455

eAppendix. Identifying Opioid-Related Overdose Events

This supplementary material has been provided by the authors to give readers additional information about their work.

© 2023 Barsky BA et al. *JAMA Health Forum*.

eAppendix. Identifying Opioid-Related Overdose Events

A. When identifying fatal and non-fatal opioid-related overdoses, we limited our study sample to overdoses within 12 months of a person's most-recent release. We identified a person's most-recent release based on Massachusetts Department of Correction (MDOC) data. These data provide individual-level incarceration records that include, among other things, the year of an individual's release from prison. If a person had multiple MDOC records, we kept the latest record and deleted the others. This procedure led us to delete 1,545 records.

B. To identify fatal opioid-related overdoses, we use data from the Massachusetts Registry of Vital Records and Statistics.

Counts are based on International Classification of Disease (ICD-10) codes and text searches of cause of death from the medical examiner's office for death records that did not have an associated ICD-10 code. The following ICD-10 codes cover deaths caused by poisonings/overdoses: X40-X49, X60-X69, X85-X90, Y10-Y19, and Y35.2. We also used fields for multiple causes of death to identify opioid-related deaths, including the following ICD-10 codes: T40.0, T40.1, T40.2, T40.3, T40.4, and T40.6.

C. To identify non-fatal opioid-related overdoses, we use data from the Massachusetts Acute Hospital Case Mix Database (Case Mix) and the Massachusetts Ambulance Trip Record Information System (MATRIS).

C.1. The Case Mix data merges include three files: (1) Hospital Inpatient Discharge Database (HD); (2) Emergency Department Database (ED); and (3) Outpatient Observation Database (OD). The HD covers discharge-level data for inpatient diagnoses. The OD covers discharge-level data for outpatient observation stays. And the ED covers visit-level data for emergency department visits.

Across all three files, we identify non-fatal opioid-related overdoses by searching for the following ICD-9 and ICD-10 diagnostic codes: "T400X1A", "T400X2A", "T400X3A", "T400X4A", "T400X1D", "T400X2D", "T400X3D", "T400X4D", "T401X1A", "T401X2A", "T401X3A", "T401X4A", "T401X1D", "T401X2D", "T401X3D", "T401X4D", "T402X1A", "T402X2A", "T402X3A", "T402X4A", "T402X1D", "T402X2D", "T402X3D", "T402X4D", "T403X1A", "T403X2A", "T403X3A", "T403X4A", "T403X1D", "T403X2D", "T403X3D", "T403X4D", "T404X1A", "T404X2A", "T404X3A", "T404X4A", "T404X1D", "T404X2D", "T404X3D", "T404X4D", "T40601A", "T40601D", "T40602A", "T40602D", "T40603A", "T40603D", "T40604A", "T40604D", "T40691A", "T40692A", "T40693A", "T40694A", "T40691D", "T40692D", "T40693D", "T40694D", "96500", "96501", "96502", "96509", "9701", "E8500", "E8501", "E8502".

We combined the records from the different datasets by date to identify duplicate records for single overdose events.

C.2. The MATRIS dataset includes data on opioid-related overdoses treated by emergency ambulance runs reported by licensed Emergency Medical Service providers. Opioid-related responses are further sorted by severity using an algorithm developed by the Massachusetts Department of Public Health. Note that when individual-level overdose events appear in both the Case Mix and MATRIS data, we remove the Case Mix event the day after MATRIS as a duplicate.

D. We have recorded opioid-related overdose deaths occurring within three days of a non-fatal opioid-related overdose as the same event (i.e., opioid-related overdose death).

© 2023 Barsky BA et al. *JAMA Health Forum*.
