# Supplementary material for: Rates of Opioid Overdose Among Racial and Ethnic Minority Individuals Released From Prison
Source: JAMA Health Forum. 2023 Dec 21;4(12):e234455. doi: 10.1001/jamahealthforum.2023.4455 (PMC10739083; doi:10.1001/jamahealthforum.2023.4455)
Supplement: Supplement 2. — Data Sharing Statement [file jamahealthforum-e234455-s002.pdf]

Data Sharing Statement

Barsky. Rates of Opioid Overdose Among Racial and Ethnic Minority Individuals Released From Prison. *JAMA Health Forum*. Published December 21, 2023.
doi:10.1001/jamahealthforum.2023.4455

Data
Data available: No
